# Supplementary material for: Single-cell mutation identification via phylogenetic inference
Source: Nat Commun. 2018 Dec 4;9:5144. doi: 10.1038/s41467-018-07627-7 (PMC6279798; doi:10.1038/s41467-018-07627-7)
Supplement: Supplementary file 1 — Supplementary Information [file 41467_2018_7627_MOESM1_ESM.pdf]

Supplementary material: Single-cell mutation identification via phylogenetic inference

Jochen Singer*, Jack Kuipers*, Katharina Jahn, Niko Beerenwinkel†

Department of Biosystems Science and Engineering, ETH Zurich, Mattenstrasse 26, 4058 Basel, Switzerland

SIB Swiss Institute of Bioinformatics, Switzerland

*These authors contributed equally.

†To whom correspondence should be addressed: niko.beerenwinkel@bsse.ethz.ch

A Probability of a mutation in k cells in a random binary tree

To compute the number of cells below a mutation in a binary genealogical tree, we work recursively. We record in $P_m(k)$ the probability of a mutation being placed uniformly among the edges of trees with m cells affecting exactly k of them. Moving from trees with m cells, which possess $(2m-1)$ edges, to trees with $(m+1)$ cells we can create a new internal node along any of the edges. This adds a further two edges. When doing so, the mutation may be above or below the new cell added, or placed along the two new edges leading to the recursion

$$P_{m+1}(k) = \frac{(2m-2k+1)}{(2m+1)}P_m(k) + \frac{(2k-3)}{(2m+1)}P_m(k-1) + \frac{\delta_{1,k}}{(2m+1)}\,, \tag{1}$$

with initial condition of $P_1(1) = 1$ and boundary conditions of $P_m(0) = 0$. The solution to the recursion in Supplementary Equation 1 is

$$P_m(k) = \frac{\binom{2k}{k}\binom{2m-2k}{m-k}}{(2k-1)\binom{2m}{m}} = \frac{\binom{m}{k}^2}{(2k-1)\binom{2m}{2k}}\,, \tag{2}$$

which can easily be shown by induction. We start with $k = 1$

$$\begin{aligned} P_{m+1}(1) =& \frac{(2m-1)}{(2m+1)}\frac{2m^2}{2m(2m-1)} + \frac{1}{(2m+1)} \\ =& \frac{1}{(2m+1)}\left[m+1\right] = \frac{2(m+1)^2}{(2m+2)(2m+1)} = \frac{\binom{m+1}{1}^2}{\binom{2m+2}{2}}\,. \end{aligned} \tag{3}$$

For $1 < k \leq m$ we have

$$\begin{aligned} P_{m+1}(k) =& \frac{(2m-2k+1)}{(2m+1)}\frac{\binom{m}{k}^2}{(2k-1)\binom{2m}{2k}} + \frac{(2k-3)}{(2m+1)}\frac{\binom{m}{k-1}^2}{(2k-3)\binom{2m}{2k-2}} \\ =& \frac{1}{(2k-1)}\frac{m!m!(2k)!(2m-2k+1)!}{k!k!(m-k)!(m-k)!(2m+1)!} \\ &+ \frac{m!m!(2k-2)!(2m-2k+2)!}{(k-1)!(k-1)!(m-k+1)!(m-k+1)!(2m+1)!}\,. \end{aligned} \tag{4}$$

Taking out a common factor, this reduces to

$$\begin{aligned} P_{m+1}(k) =& \frac{2m!m!(2k-2)!(2m-2k+1)!}{(k-1)!(k-1)!(m-k)!(m-k)!(2m+1)!}\left[\frac{1}{k} + \frac{1}{m-k+1}\right] \\ =& \frac{2m!(m+1)!(2k-2)!(2m-2k+1)!}{(k-1)!k!(m-k)!(m-k+1)!(2m+1)!} \\ =& \frac{(m+1)!(m+1)!(2k)!(2m-2k+2)!}{(2k-1)!k!k!(m-k+1)!(m-k+1)!(2m+2)!} = \frac{\binom{m+1}{k}^2}{(2k-1)\binom{2m+2}{2k}}\,. \end{aligned} \tag{5}$$

B Efficient likelihood computation for wild type loci

The beta-binomial density function of Equation 1 can be expressed using Gamma functions as:

$$P(s \mid c,\, \alpha,\, \beta) = \frac{\Gamma(c+1)}{\Gamma(s+1)\Gamma(c-s+1)}\frac{\Gamma(s+\alpha)\Gamma(c-s+\beta)}{\Gamma(c+\alpha+\beta)}\frac{\Gamma(\alpha+\beta)}{\Gamma(\alpha)\Gamma(\beta)} \tag{6}$$

For numerical accuracy we compute log probabilities such that the factors become summands. These can be treated individually, leading to nine terms per locus. However, summands only involving s and c depend solely on the data and not on the tree or parameters. As they do not change between iterations of the algorithm, they can be ignored. The summands involving only α and β do not depend on the locus and can be computed in $O(1)$ for all loci. This leaves us with three summands that need to be recomputed whenever one of the parameters of the model changes. For efficiency, we store an array with occurrences of unique values of s, $c - s$ and c. This reduces the time complexity from $O(Nm)$ to $O(c)$ for the computation of the probability of all $(N - n)$ wild type loci.

C Variant calling pipeline

In order to reliably call mutations, we applied several mapping and purification steps using NGS-pipe [1]. We first mapped the downloaded FASTQ files using BWA-mem [2] version 0.7.15 to the human reference genome $hg19$. The resulting files were merged, sorted and duplicates removed (for exome data) using Picard tools (http://broadinstitute.github.io/picard/) version 2.8.3. Afterwards we realigned the reads around indels using the GATK [3] version 3.5. SAMtools mpileup [4] version 1.3.1 was used with parameters `-A -B -d 1000 -q 40 -Q 30` for the exome dataset and `-A -B -d 100000 -q 40 -Q 30` for the panel dataset. Monovar (commit 7b47571) was then run according to the authors' recommendation (https://bitbucket.org/hamimzafar/monovar). In order to compare the results, the normalized and Phred-scaled likelihoods for the genotypes reported by Monovar were back-transformed and the fraction of heterozygous plus homozygous genotype likelihoods reported as the probability of the mutation. Further, hierarchical clustering of Monovar mutation calls was performed in R [5] version 3.4.3 using the $heatmap.2$ function and assigning 0.5 in case of missing information. SCIΦ was run with default values with an additional filter of requiring at least two cells to show an alternative nucleotide count of at least three. For the exome data set we used the regions specified by the Nextera Exome Enrichment Kit from Illumina and one million iterations of the MCMC scheme. For the panel data set we used loci indicated as likely mutated as described in [6], based on bulk sequencing. In addition, for the panel data we first used SCIΦ without parameter learning for 500.000 iterations and used the result as a starting tree for the final run with five million iterations.

Further, variants with low VAFs across all cells for a given locus where filtered out by SCIΦ using a likelihood ratio test. In order to do so, we first fitted a beta-binomial distribution with free mean and overdispersion to maximise the likelihood of that locus across all cells with non-zero variant reads and computed the likelihood L_1. For the same set of cells, a second beta-binomial distribution with a mean fixed to 0.25 (to allow for copy number changes) and free overdispersion was fitted to compute the maximum likelihood L_0 of the constrained model. The test statistic $-2 \log \frac{L_1}{L_0}$ is asymptotically χ^2_1 distributed and loci with a p-value > 0.05 or estimated mean > 0.25 were kept. For the exome data, loci with coverage less than six in the control bulk sequencing dataset were excluded as not reliably distinguishable from germline variants. Further, loci showing an alternative nucleotide count of 2 or more in the bulk control were excluded as germline variants.

MDA errors can rarely lead to high VAFs at wildtype positions. For high coverage data, such as panel sequencing data sets, such false positives would have extremely low probabilities under the wild type model which only accounts for sequencing errors (Equation 2). SCIΦ would then falsely assign the mutations to the cells. In addition, the false positive calls may then be assigned to all neighboring cells of the clade. To account for the possibility of high VAF false positives in high

coverage panel sequencing data sets, we therefore defined

$$P_{\mathrm{wt}}(D_{ij}) = (1-\mathrm{s})P\left(s_{ij} \mid c_{ij},\, f_{\mathrm{wt}},\, \omega_{\mathrm{wt}}\right) + \mathrm{s}P\left(s_{ij} \mid c_{ij},\, 0.5 - \frac{2}{3}f_{\mathrm{wt}},\, \omega_{\mathrm{a}}\right), \tag{7}$$

with s being the MDA substitution error set to 5×10^{-7}.

D Influence of prior parameters

In an additional experiment we investigated the influence of changing the prior parameters for SCIΦ and Monovar (Supplementary Figure 1). For SCIΦ we changed the prior probability λ of a locus to be mutated, where the default is 0.0001. For Monovar we investigated the influence of the prior probability of a false-positive error.

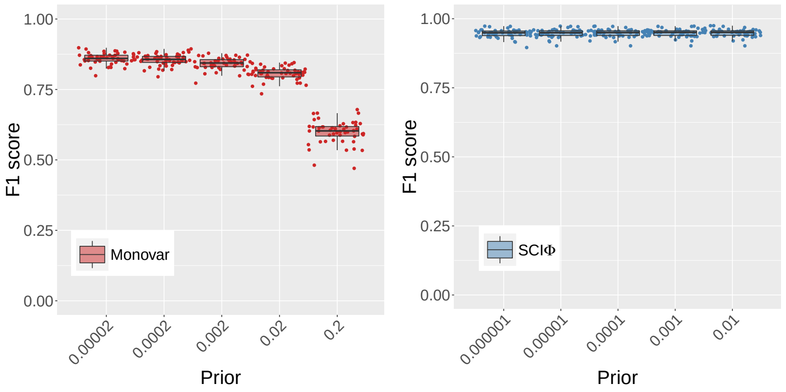

Supplementary Figure 1: Summary statistics of F1 performance from (a) Monovar and (b) SCIΦ on simulated data as their prior parameters are varied.

The F1 score does not change dramatically over five orders of magnitude (except with a very high prior for Monovar).

E Results for alternative simulation

In contrast to the previously described simulation setting, here we simulate a dependence between the homologous chromosomes, as has been observed, e.g., in [7]. The initial number of alleles is changed from 1 to 2 for both α and β. A biological explanation for doing so is the observation that amplification templates can only occur as a multiples of 2, due to the double stranded arrangement of the DNA. While heterozygous positions contain two chromosomes, one with two wild type strands and one with two mutant strands ($\alpha = 2, \beta = 2$), drop-out positions only retain the two strands of one of the two chromosomes ($\alpha = 2, \beta = 0$; $\alpha = 0, \beta = 2$,). The results are similar to the previous simulation and summarized in Supplementary Figure 2.

In addition, Supplementary Figure 3 replicates the performance comparison of Section 2.3 with the difference of also including loci with missing information into the performance evaluation of SCIΦ. The results are very similar to the case where only loci with coverage were considered (Section 2.3), demonstrating the capability of SCIΦ to predict accurate mutation calls even in case of missing information.

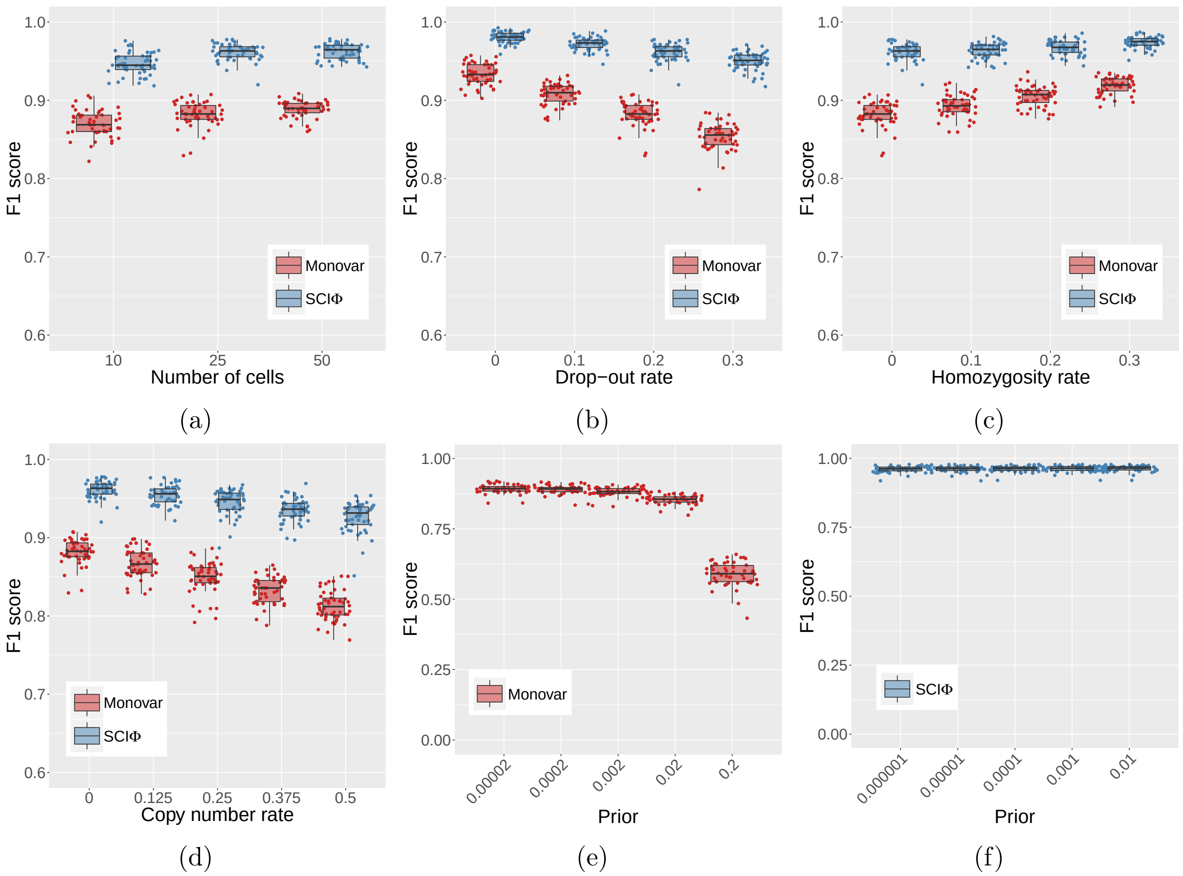

Supplementary Figure 2: Summary statistics of the F1 performance from Monovar and SCIΦ on simulated data with α and β set to 2 for the Pólya urn MDA process. F1 score depending on (a) the number of cells, (b) the drop-out rate, (c) the homozygosity rate, (d) the copy number rate, (e) the false positive prior for Monovar, and (f) the mutation prior for SCIΦ.

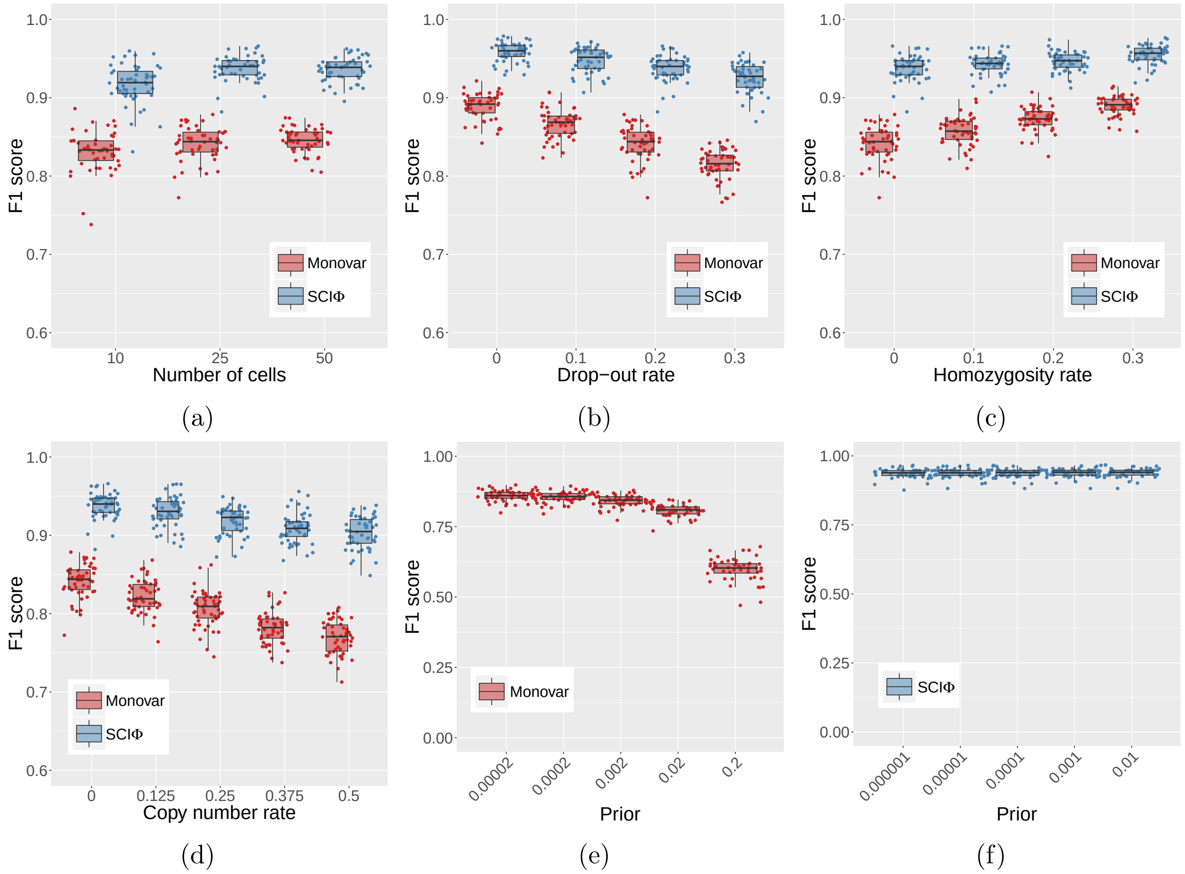

Supplementary Figure 3: Summary statistics of the F1 performance from Monovar and SCIΦ on simulated data with α and β set to 1 for the Pólya urn MDA process. In contrast to Monovar, which does not provide a genotype for loci with missing information, all SCIΦ genotypes, including loci with missing information, are included in this benchmark. F1 score depending on (a) the number of cells, (b) the drop-out rate, (c) the homozygosity rate, (d) the copy number rate, (e) the false positive prior for Monovar, and (f) the mutation prior for SCIΦ.

F Influence of the amplification process

Due to the limited amount of initial genomic material, it has to be amplified extensively. The most common approach for this is isothermal Multiple Displacement Amplification (MDA) [8]. Extensive validations using simulated data to mimic MDA with various parameterizations have been conducted in Section 4.8 and Supplementary Section E. However, there are other protocols purely based on PCR amplification or initial isothermal preamplification followed by PCR amplification [9]. In order to investigate how the performance of Monovar and SCIΦ change with different amplification schemes we used the previously described simulation framework and varied α and β, representing the initial copies of the homologues chromosomes. With increasing α and β, the simulation becomes increasingly similar to traditional bulk sequencing data sets with more initial genomic material. SCIΦ as well as Monovar show increasing F1 measures with increasing initial genetic material (Supplementary Figure 4).

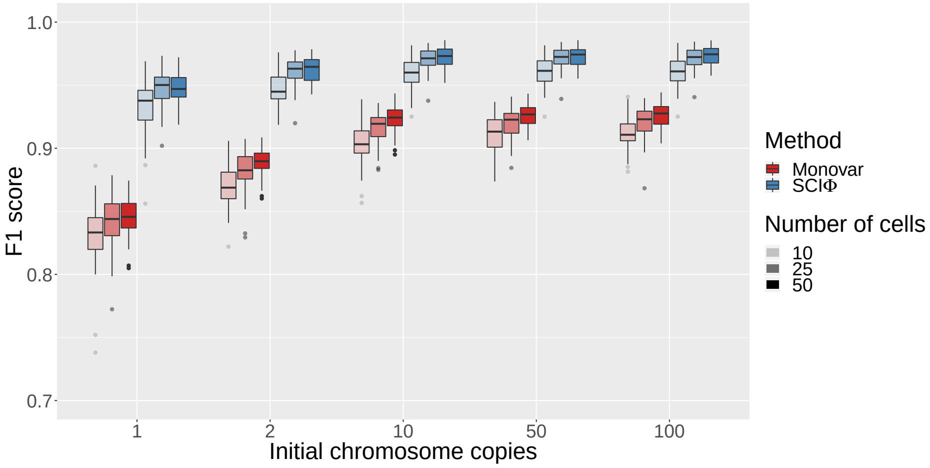

Supplementary Figure 4: Graphical representation of the influence of the amplification process on the F1 performance measure. The F1 score was measured with respect to different number of cells, ranging from 10 to 50, and different numbers of initial copies of the homologues chromosomes, ranging from 1 copy of maternal and paternal chromosome to 100 initial copies each.

G Comparison of single-cell and bulk sequencing variant callers

Bulk sequencing variant calling approaches are-ill suited to identify mutations in single cell data [10]. We compared SCIΦ and Monovar to VarScan2 [11] (version 2.4.3) with default parameters. The drop-out rate of the experiment was changed, as this is one of the distinguishing features of single-cell sequencing data sets. The results confirm the increased performance of specialized single cell variant callers compared to VarScan2 (Supplementary Figure 5).

H Violation of the infinite site assumption

A central assumption underlying SCIΦ is the infinite site assumption, which states that a mutation can only occur once in the tree and is never lost afterwards. This assumption might not hold in all tumors [12]. Therefore, we investigated the influence of breaking the infinite site assumption. In order to do so, we randomly introduce a fraction of mutations recurring in different branches as well

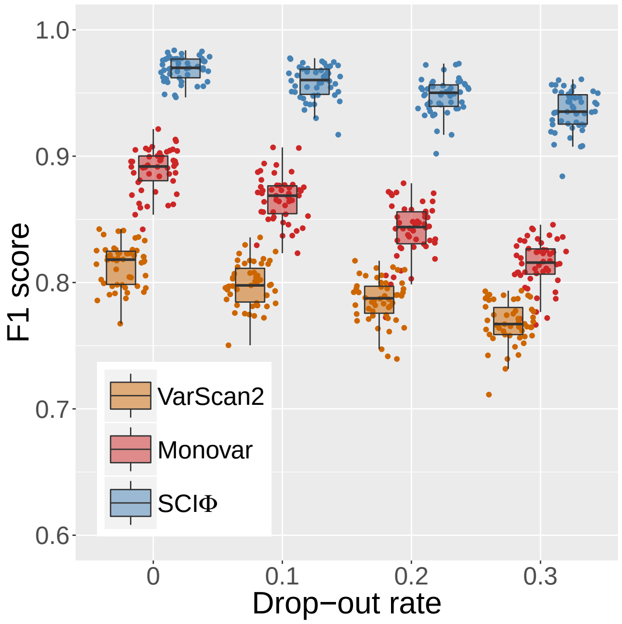

Supplementary Figure 5: Performance of VarScan2 in comparison to SCIΦ and Monovar in terms of the F1 measure with varying drop-out rate.

as mutations lost in a subtree. As expected, the performance of SCIΦ decreases with increasing number of violations of the infinite site assumption (Supplementary Figure 6). While the recall is steady the precision changes. In the case of convergent evolution the mutation is assigned to the lowest common ancestor and the missing evidence for the cells without the mutation is explained via drop-out events. In the case of a mutation loss in a subtree, the algorithm will explain the missing evidence in the subtree with drop-out events again. However, even though mutations are wrongly assigned, the tree structure is stably inferred, since the sensitivity is unaffected.

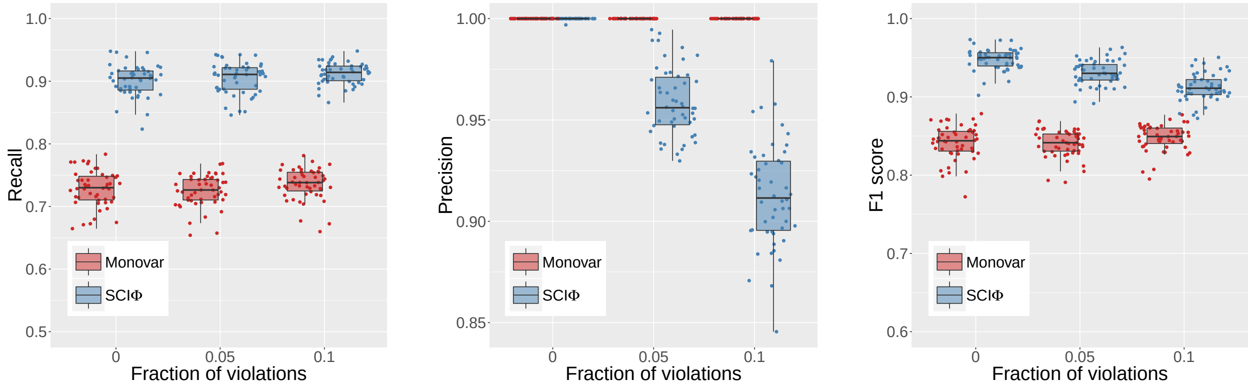

(a) Recall (b) Precision (c) F1 score

Supplementary Figure 6: Summary statistics of the performance of SCIΦ and Monovar on simulated data with different levels infinite site violations.

I Tree inference performance

In order to analyze the tree inference performance of SCIΦ we investigated the similarity of the tree inferred by SCIΦ and the corresponding simulated tree. The distance measure we chose is closely related to the Frobenius norm and takes into consideration that differences in the lower parts of the tree, close to the leaves, do not influence as many mutations as differences close to the root. For each pair of cells, we separately compute how distant the genotypes are in the ground truth and inferred models and then compute the squared difference between these distances. The distance between the ground truth tree and the tree inferred by SCIΦ is then defined as:

$$\sqrt{\frac{2}{m(m-1)}\frac{1}{n}\sum_{i=1}^{m}\sum_{j=i+1}^{m}\sum_{k=1}^{n}\left(|\mathrm{M}_{i,k}-\mathrm{M}_{j,k}|-|\mathrm{M}'_{i,k}-\mathrm{M}'_{j,k}|\right)^2}, \tag{8}$$

where m is the number of cells, n the number of identified mutations, and M and M′ are the probabilities of the mutations in the ground truth (0 or 1) and the probabilities of the mutations reported by SCIΦ, with $\mathrm{M'}_{i,k}$ the probability of the k-th inferred mutation in cell i. In order to provide a reference point we also compute the distance between two random trees of the same size. The distance of the inferred trees to the ground truth trees is always much smaller than the distance of random trees (Supplementary Figure 7).

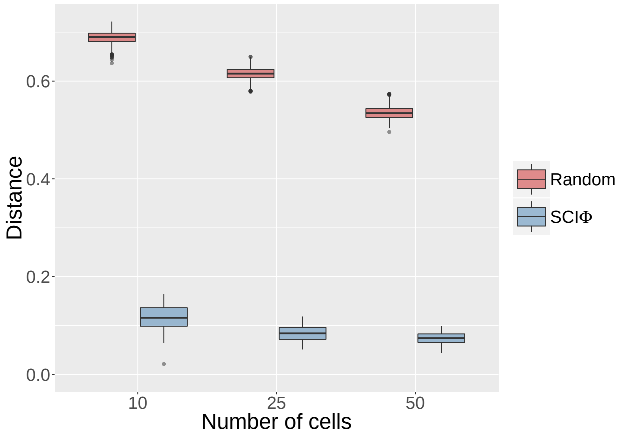

Supplementary Figure 7: Tree distances between random trees and trees inferred by SCIΦ and its corresponding ground truth tree.

J Performance comparison using isogenic cell line data

Because simulated data can only be an approximation of real data, we compared the performance of Monovar and SCIΦ on a isogenic fibroblast cell line consisting of 19 single cells and a reference bulk sample, described in [13] (Sequence Read Archive accession number SRP046355). Because the single cells are genetically very similar we can use the reference bulk sample to define a set of ground truth variants to compare against. For this, we used the pipeline described in Supplementary Section C and applied GATK HaplotypeCaller [14] (version 3.8-0) to the reference sample with parameters -mmq 40 -mbq 30. Afterwards we used GATK GenotypeGVCFs [14] (version 3.8-0) to obtain mutations. The final ground truth set was then created by filtering for SNVs and loci with a coverage of at least 30x. Monovar and SCIΦ were run as described earlier, requiring at least two cells

with an alternative support of three. Results for the mutation calls are provided in Supplementary Table 1.

	True Positives	False Negatives	False Positives	Recall	Precision	F1
Monovar	108942	21885	32402	0.83	0.77	0.80
SCIΦ	122892	7935	42274	0.94	0.74	0.83

Supplementary Table 1: Summary statistics of identified mutations by Monovar and SCIΦ.

In the comparison, SCIΦ shows a higher F1-score and recall. In terms of precision Monovar achieves a higher score. With respect to precision, Monovar as well as SCIΦ only achieve 77% and 74%, respectively. However, investigating the nucleotide distributions for supposedly false positive mutation calls revealed strong evidence for mutations to be present. Supplementary Figure 8 shows the percentages of cells with a coverage of five or higher and with at least 50% mutation supporting reads for loci containing false positive calls of Monovar and SCIΦ. Most loci that were declared wild type by GATK HaplotypeCaller contain several cells with at least 50% alternative supporting reads. Therefore, we conclude that many of the false positive calls should be classified as true positives.

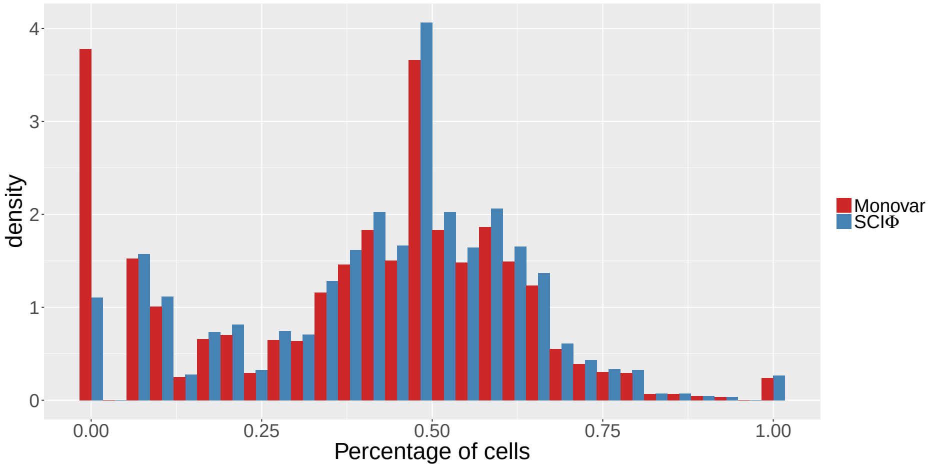

Supplementary Figure 8: Histogram showing the percentage of cells per locus with at least 50% mutation supporting reads.

This data set is particularly challenging for SCIΦ as we do not expect a pronounced phylogenetic structure within an isogenic cell line. Even in this situation the inference scheme works well and assigns the majority of the mutations to the root (Supplementary Figure 9). The observed linear structure of the tree is a result of sorting the drop-out events. However, most of the mutations are assigned to nodes close to the root, such that they are identified in almost all cells.

K Performance comparison on a high-grade serous ovarian cancer patient

In addition to the panel data set described in Section 2.4, we also analyzed data from 370 cells of a high-grade serous ovarian cancer patient described in [15]. We extracted the nucleotide counts for the targeted loci from the supplementary material of [15] for cells with more than 10.000 reads. Our inferred tree (Supplementary Figure 10) shows a very similar structure compared to the original

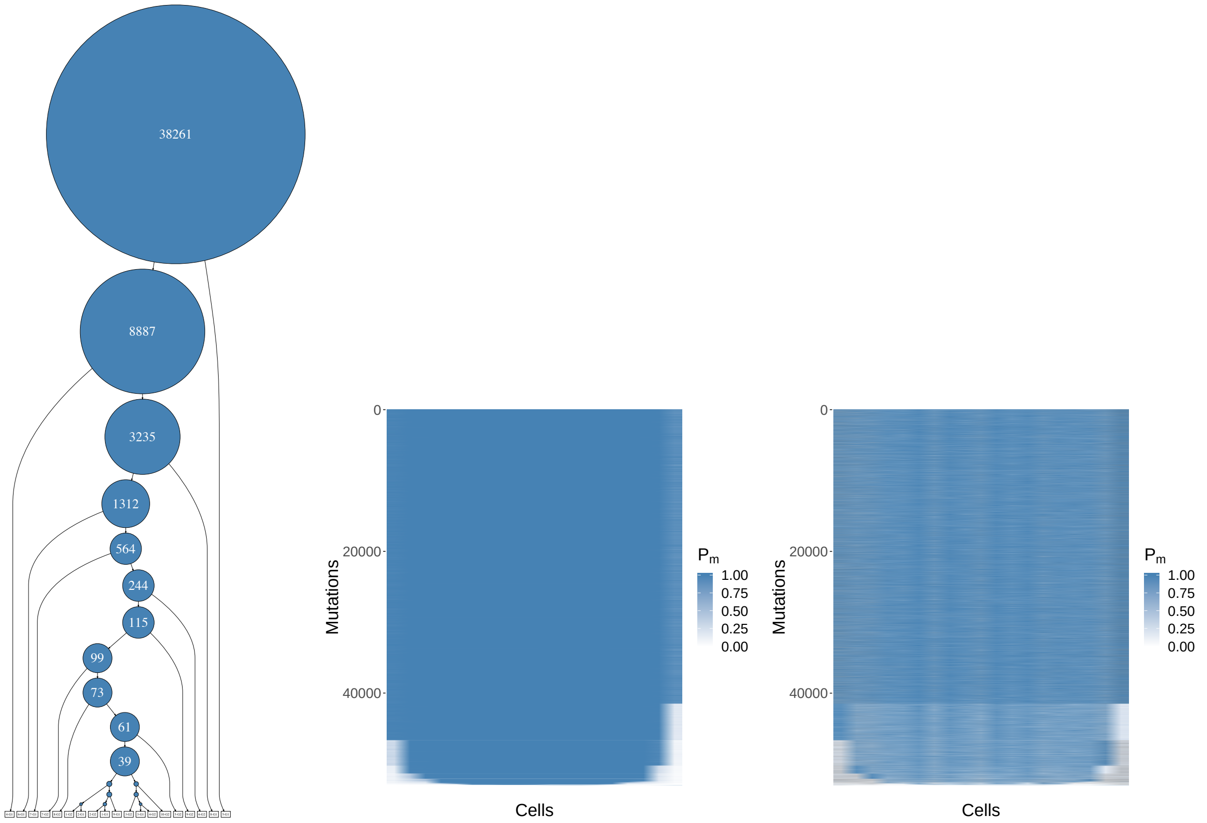

(a) (b) (c)

Supplementary Figure 9: Summary of the mutation calls from SCIΦ and Monovar on a isogenic fibroblast cell line consisting of 19 single cells and a reference bulk sample, described in [13]. (a) Maximum a posteriori tree of SCIΦ with the number of mutations per node. (b) SCIΦ and (c) Monovar mutation to node assignments.

publication, including a cluster of normal cells. Interestingly, Monovar assigns the mutation in lines 9 and 25 of Supplementary Figure 10 also to the normal cells. A closer investigation of these loci revealed that their VAFs were always below 0.008 except for three mutations. Two of them had a VAF of 0.31 and 0.33, however, their coverage where only 19 and 3, while the remaining loci had coverages in the thousands. Only one mutation had a coverage of 5689 and alternative support of 2281. However, it is more likely that this is an error early in the amplification rather than all other normal cells showing a VAF below 1% in the presence of a mutation. In contrast, the mutation in the first row shows five cells with VAFs greater than 12%. Therefore, SCIΦ imputes the presence of the mutation in all cells. Since the remaining normal cells show VAFs below 1%, it remains unclear whether this is a true mutation or a false positive call for Monovar and SCIΦ for the normal cells.

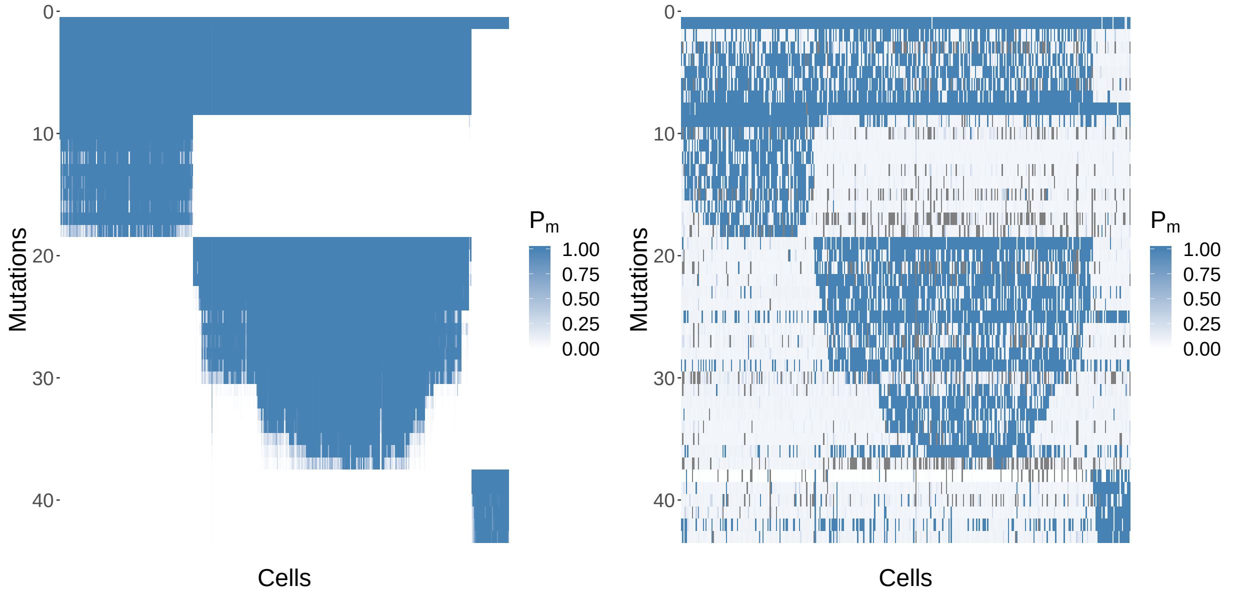

(a) (b)

Supplementary Figure 10: Mutation to cell assignments for (a) SCIΦ and (b) Monovar for a high-grade serous ovarian cancers patient described in [15].

L Computational resources.

Here we provide run times and memory consumption peaks for Monovar and SCIΦ on the benchmarked real data sets. All runs finished within hours and could be run on a typical desktop PC.

			Monovar		SCIΦ	
sample	seq type	# cells	time	mem	time	mem
triple-negative breast cancer	exome	16	98min	64MB	160min	2GB
isogenic fibroblast cell line	exome	19	3.2h	73MB	11.3h	4GB
acute lymphoblastic leukemia	panel	255	34.9min	114MB	12.5h	120MB
high-grade serous ovarian	panel	370	88.1min	1169MB	12.8h	202MB

Supplementary Table 2: Summary statistics of identified mutations by Monovar and SCIΦ

M Supplementary figures

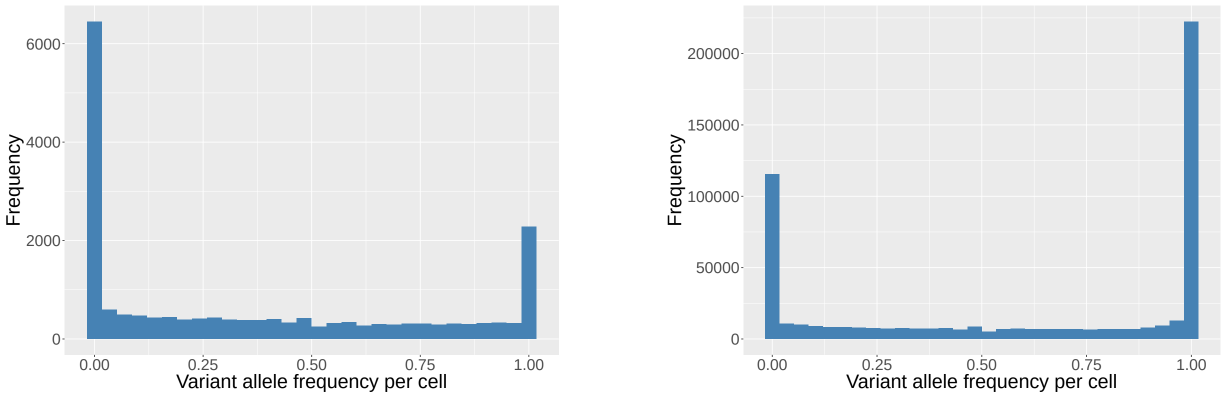

(a) (b)

Supplementary Figure 11: Variant allele frequencies (VAFs) of the single cells for positions containing somatic mutations identified by SCIΦ (a) and positions with differences to the reference genome, including germline variants, identified by Monovar (b) in the data described in [16]. Only entries with coverage of at least 10 are shown to circumvent coverage artifacts.

N Supplementary tables

Parameter	Description
f_{wt}	Sequencing error rate: Parameter used in the beta-binomial model of the wild type nucleotide counts
ω_{wt}	Shape parameter: Parameter used in the beta-binomial model of the wild type nucleotide counts to model the shape of the distribution
ω_{a}	Shape parameter: Parameter used in the beta-binomial model of the alternative nucleotide counts to model the shape of the distribution
μ	Drop-out rate: Parameter describing the fraction of mutations affected by drop-outs
ν	Homozygosity rate: Parameter describing the fraction of mutations that are homozygous alternative.

Supplementary Table 3: Model parameters and their description

References

[1] Singer, J. *et al.* NGS-pipe: a flexible, easily extendable and highly configurable framework for NGS analysis. *Bioinformatics* **34**, 107–108 (2017).

[2] Li, H. & Durbin, R. Fast and accurate short read alignment with Burrows–Wheeler transform. *Bioinformatics* **25**, 1754–1760 (2009).

[3] McKenna, A. *et al.* The genome analysis toolkit: a MapReduce framework for analyzing next-generation DNA sequencing data. *Genome Res.* **20**, 1297–1303 (2010).

[4] Li, H. *et al.* The sequence alignment/map format and SAMtools. *Bioinformatics* **25**, 2078–2079 (2009).

[5] R Development Core Team. *R: A Language and Environment for Statistical Computing*. R Foundation for Statistical Computing, Vienna, Austria (2008). ISBN 3-900051-07-0.

[6] Gawad, C., Koh, W. & Quake, S. R. Dissecting the clonal origins of childhood acute lymphoblastic leukemia by single-cell genomics. *Proc. Natl. Acad. Sci. U.S.A.* **111**, 17947–17952 (2014).

[7] Lodato, M. A. *et al.* Somatic mutation in single human neurons tracks developmental and transcriptional history. *Science* **350**, 94–98 (2015).

[8] Lasken, R. S. Genomic DNA amplification by the multiple displacement amplification (MDA) method. *Biochem. Soc. Trans.* **37**, 450–453 (2009).

[9] Gawad, C., Koh, W. & Quake, S. R. Single-cell genome sequencing: current state of the science. *Nat. Rev. Genet.* **17**, 175–188 (2016).

[10] Zafar, H., Wang, Y., Nakhleh, L., Navin, N. & Chen, K. Monovar: single-nucleotide variant detection in single cells. *Nat. Methods* **13**, 505–507 (2016).

[11] Koboldt, D. C. *et al.* VarScan 2: Somatic mutation and copy number alteration discovery in cancer by exome sequencing. *Genome Res.* **22**, 568–576 (2012).

[12] Kuipers, J., Jahn, K., Raphael, B. J. & Beerenwinkel, N. Single-cell sequencing data reveal widespread recurrence and loss of mutational hits in the life histories of tumors. *Genome Res.* **27**, 1885–1894 (2017).

[13] Leung, M. L., Wang, Y., Waters, J. & Navin, N. E. SNES: single nucleus exome sequencing. *Genome Biol.* **16**, 55 (2015).

[14] Van der Auwera, G. A. *et al.* From FastQ data to high-confidence variant calls: the genome analysis toolkit best practices pipeline. *Curr. Protoc. Bioinformatics* **43**, 11–10 (2013).

[15] McPherson, A. *et al.* Divergent modes of clonal spread and intraperitoneal mixing in high-grade serous ovarian cancer. *Nat. Genet.* **48**, 758–767 (2016).

[16] Wang, Y. *et al.* Clonal evolution in breast cancer revealed by single nucleus genome sequencing. *Nature* **512**, 155–160 (2014).
